# A study on trypsin, *Aspergillus flavus *and *Bacillus *sp. protease inhibitory activity in *Cassia tora *(L.) syn *Senna tora *(L.) Roxb. seed extract

**DOI:** 10.1186/1472-6882-11-56

**Published:** 2011-07-12

**Authors:** Vinayak R Tripathi, Shailendra Kumar, Satyendra K Garg

**Affiliations:** 1 Centre of Excellence in Microbiology, Dr. Ram Manohar Lohia Avadh University, Faizabad-224001, Uttar Pradesh, India

## Abstract

**Background:**

Proteases play an important role in virulence of many human, plant and insect pathogens. The proteinaceous protease inhibitors of plant origin have been reported widely from many plant species. The inhibitors may potentially be used for multiple therapeutic applications in viral, bacterial, fungal diseases and physiological disorders. In traditional Indian medicine system, *Cassia tora *(*Senna tora*) is reportedly effective in treatment of skin and gastrointestinal disorders. The present study explores the protease inhibitory activity of the above plant seeds against trypsin, *Aspergillus flavus *and *Bacillus *sp. proteases.

**Methods:**

The crushed seeds of *Cassia tora *were washed thoroughly with acetone and hexane for depigmentation and defatting. The proteins were fractionated by ammonium sulphate (0-30, 30-60, 60-90%) followed by dialysis and size exclusion chromatography (SEC). The inhibitory potential of crude seed extract and most active dialyzed fraction against trypsin and proteases was established by spot test using unprocessed x-ray film and casein digestion methods, respectively. Electrophoretic analysis of most active fraction (30-60%) and SEC elutes were carried employing Sodium dodecyl sulphate polyacrylamide gel electrophoresis (SDS-PAGE) and Gelatin SDS-PAGE. Inhibition of fungal spore germination was studied in the presence of dialyzed active inhibitor fraction. Standard deviation (SD) and ANOVA were employed as statistical tools.

**Results:**

The crude seeds' extract displayed strong antitryptic, bacterial and fungal protease inhibitory activity on x-ray film. The seed protein fraction 30-60% was found most active for trypsin inhibition in caseinolytic assay (P < 0.001). The inhibition of caseinolytic activity of the proteases increased with increasing ratio of seed extract. The residual activity of trypsin, *Aspergillus flavus *and *Bacillus *sp. proteases remained only 4, 7 and 3.1%, respectively when proteases were incubated with 3 mg ml^-1 ^seed protein extract for 60 min. The inhibitory activity was evident in gelatin SDS-PAGE where a major band (~17-19 kD) of protease inhibitor (PI) was detected in dialyzed and SEC elute. The conidial germination of *Aspergillus flavus *was moderately inhibited (30%) by the dialyzed seed extract.

**Conclusions:**

*Cassia tora *seed extract has strong protease inhibitory activity against trypsin, *Aspergillus flavus *and *Bacillus *sp. proteases. The inhibitor in *Cassia tora *may attenuate microbial proteases and also might be used as phytoprotecting agent.

## Background

Proteases constitute one of the largest functional group of proteins involved in many normal and pathological processes. Protease inhibition of pathogenic organisms may aid in control of several diseases [1]. Plants are known to synthesize self-protective compounds as well as accumulate inducible proteins that can directly affect microbes [2]. A number of such antifungal and antibacterial proteins include lectins, ribosomal inactivating proteins, zeamatin, chitinases, glucanases, thionins and protease inhibitors [2]. Recently, inhibitors of proteases have shown promise in their emergence as alternative approach in antiviral, antibacterial to anticarcinogenic treatments [1,3]. The advent of recent biotechnological and pharmaceutical approaches envisages application of protease inhibitors to contain plant and human diseases. Traditional Indian medicinal system emphasizes the use of plants and herbs for many maladies. Most of these are used without much knowledge about their active constituents and mechanism of action. *Cassia tora *(*Senna tora*), a member of Leguminosae (subfamily Caesalpinoideae), is found as weed throughout the India. The herb is reportedly beneficial in skin diseases, possesses anthelmintic properties and at times recommended in liver complaints and gastrointestinal disorders [4].

Proteinaceous protease inhibitors are important defense molecules expressed in various plants, animals and microbes [5]. The evidence of protease inhibitors' involvement in plant defense was demonstrated as early in 1947 by Mickel and Standish, when they observed that the larvae of certain insects were unable to develop normally on soybean products [6]. Subsequently, the trypsin inhibitors present in soybean were shown to be toxic to the larvae of flour beetle, *Tribolium confusum *[7]. These findings were further substantiated by expression of the cowpea trypsin inhibitor gene in tobacco, which increased its resistance against herbivorous insects [8]. The plant protease inhibitors are generally small proteins, which regulate significant physiological processes, and are also induced upon attack by insects or pathogens [9]. Protease inhibitors exhibit a peculiar property of forming complexes with proteolytic enzymes and promote inhibition of their activity by competing for the catalytic site. Majority of proteinase inhibitors studied in plant kingdom originate from three main families namely leguminosae, solanaceae and gramineae [10].

*Aspergillus *sp. is a causative agent of different diseases of plants, humans, insects and other organisms [11]. Aspergilli are commonly considered as opportunistic pathogens. *Aspergillus flavus *is a unique species as it has wide host range from humans, plants, insects to animals, and produces one of the potentially harmful aflatoxins. Aflatoxins are mycotoxins produced as secondary metabolites by aspergilli and not known to have any specific biological role in these organisms [12]. It is widely distributed in tropical and subtropical zones around the globe. *Aspergillus flavus *isolates obtained from cottonseed, corn, peanut, insect and human display proteinase activity to various extents [13]. They secrete variety of proteinases but major appears to be serine and metallo-proteinases [14]. On account of aflatoxin B1 production, it assumed significant agricultural importance. Crops affected by aflatoxins include cotton, peanut, tree nut, corn (maize), rice, pepper, figure and spices. When *Aspergillus flavus *infects susceptible crops, contamination with aflatoxin greatly reduces the value of the commodity [15]. They also become harmful for animals by their aflatoxins, which cause mortality and reduced productivity in farm animals.

The aflatoxins are detrimental to humans, as high concentrations have been associated with liver cancer [12]. Further, *Aspergillus flavus *has been associated with secondary respiratory infections in immuno-compromised patients. *Aspergillus flavus *is considered to be second leading cause of invasive aspergillosis [16].

*Bacillus *species is widely distributed in nature. It has been reported from nosocomial environments as well as from soil, water and air. Normally they are considered avirulent, but three species such as *Bacillus cereus*, *Bacillus anthracis *and *Bacillus thuringiensis *have received wide attention [17]. While *Bacillus anthracis *is responsible for anthrax, *Bacillus thuringiensis *is known for its insecticidal toxins. *Bacillus cereus *is reported in food poisoning, and more severely for endopthalamitis, meningoencephalitis and infection in immunocompromised patients [17-20]. The proteases along with enterotoxins, emetic toxin (cereulide), hemolysins and phoshpolipase C as well as other enzymes like β-lactamases and collagenases from *Bacillus *sp. are known potential virulence factors [21].

In the present study, protease inhibitory activity of *Cassia tora *seed extract has been investigated against trypsin and proteases of *Bacillus *sp. isolate and pathogenic fungus *Aspergillus flavus *employing spot test method using unprocessed x-ray film and caseinolytic assay. Electrophoretic (SDS and Gelatin- SDS PAGE) analysis is carried out to confirm the hitherto unreported trypsin resistant proteinaceous protease inhibitory activity in seed. Effect of seed extract on *Aspergillus flavus *spore germination has also been studied.

## Methods

### Plant material and extraction of protease inhibitor (PI)

The *Cassia tora *seeds were collected from fields of Faizabad region (UP, India) during November 2008 to January 2009. The plant seeds were identified as *Cassia tora *L. Syn. *Senna tora *(L.) Roxb. and deposited at Raw Materials Herbarium & Museum, NISCAIR, New Delhi (specimen reference number 1729/29). The seed extract was prepared as per the method described by Mulimani *et al*. [22]. Briefly, 100 gram dry seeds of *Cassia tora *were finely powdered, depigmented and defatted with 3 volumes of chilled acetone washing and allowed to air dry. Further washing was carried out with 2 volumes of chilled hexane. After complete air drying, the seed powder was soaked overnight in 100 mM sodium phosphate buffer (pH 7.1) at 4°C (containing 1% polyvinyl pyrrolidine for effective removal of phenols). The suspension was centrifuged at 12,000 g for 20 min at 4°C. The supernatant was used as a source of crude plant extract. Further, supernatant was saturated with ammonium sulphate in three stages, i.e., 0 -30%, 30-60% and 60-90% for precipitation of proteins present. Protein pellet obtained in each step was dissolved in minimum volume of 100 mM sodium phosphate buffer (pH 7.1), and dialyzed extensively against water using 12 kD membrane (Sigma chemicals) for 24 h. For inhibitory activity analyses, the seeds' endoproteolytic and protease inhibitory activity were inactivated by heating at optimized 55°C for 15 min and 100°C for 60 min, respectively.

Each dialyzed fraction was analyzed using caseinolytic method [23] for its PI activity and 500 μl of most active fraction (30-60%) was subjected to size exclusion chromatography using Sephadex G-75 (Sigma chemicals) gel filtration column (20 × 300 mm). Prewashing of column was done with 100 ml of the Tris-HCl buffer (pH 8.0, 50 mM). The proteins were eluted in 2 ml fractions using same buffer and each fraction was analyzed at 280 nm to detect the presence of proteins. The protein was quantified as per Lowry *et al*. [24]. Activity of each fraction was estimated and active fractions were pooled [23].

### Proteases of bacterial, fungal and animal origin

A bacterium was isolated from the local district hospital ward, and identified as *Bacillus *sp. employing cultural and biochemical characteristics as per the Bergey's Manual of Systematic Bacteriology [25]. It was grown in modified Glucose Yeast Extract (GYE) broth containing (gl^-1 ^distilled water): glucose, 10.0; peptone, 10.0; yeast extract, 5.0 and NaCl, 5.0 [26]. For protease production, a loopful culture was inoculated in 100 ml modified GYE broth (pH 9; adjusted using sterilized 1 M Na_2_CO_3 _solution after autoclaving) in Erlenmeyer flasks and incubated at 37 ± 1°C on incubator shaker (150 rpm) for 20 h. After incubation, culture broth was centrifuged at 16,000 g (4°C) for 5 min and cell-free supernatant was used as protease source. *Aspergillus flavus *(MTCC 2798) was procured from the Microbial Type Culture Collection, Institute of Microbial Technology, Chandigarh, India. The fungal culture was maintained on 5 g solid substrate (2.5 g wheat bran + 2.5 g corn cob) moistened with 10 ml of moistening medium containing (gl^-1 ^distilled water): glucose, 10.0; peptone, 10.0; malt extract, 5.0 and CaCl_2_, 0.1. After complete sporulation, the culture was soaked in 25 ml sterile distilled water, filtered after 4 h and the filtrate was taken as a source of enzyme. Bovine trypsin (EC. 3.4.21.4) from Himedia Laboratories Pvt. Ltd., India was used directly as an animal protease.

### Protease assay

The protease activity was assayed by the casein digestion method [23]. One ml of enzyme was incubated with 3.0 ml of 1% (w/v) casein (prepared in 100 mM Tris-HCl buffer; pH 8.0) at 37 ± 1°C, and after 10 min, the reaction was stopped by addition of 3.0 ml of 10% (w/v) trichloroacetic acid (TCA). The mixture was centrifuged at 16,000 g for 10 min, and absorbance of supernatant was taken at 275 nm to estimate the released tyrosine using tyrosine as standard. One protease unit was defined as the amount of enzyme that liberates 1.0 μg of tyrosine min^-1 ^ml^-1 ^[23].

### Detection of protease inhibitory (PI) activity

#### (A) Spot-test analysis

Twenty μl of trypsin (5, 10 and 15%, w/v in Tris-HCl buffer, pH 8.0) was spotted on to gelatin coated x-ray film (Kodak) and incubated at 37°C for 30 min to observe gelatin digestion. The crude extract was diluted suitably with Tris-HCl buffer (pH 8.0) to get 3 different protein concentrations of 2.0, 5.0 and 12.5 μg ml^-1^. Each dilution was incubated with equal volume of trypsin (10%, w/v) at 37°C for 60 min and applied on x-ray film as described previously. The film was washed using mildly warm water to observe the extent of gelatin clearance. The seed extracts with and without buffer were spotted as controls. Similar reactions were carried out using proteases of *Aspergillus flavus *and *Bacillus *sp., instead of trypsin. Later, the presence of protease inhibitory activity of seed was confirmed using ammonium sulphate precipitated fractions and elutes of SEC.

#### (B) Casein digestion (Caseinolytic) method

Equal volume of ammonium sulphate fractions (0-30, 30-60, 60-90%) and trypsin (10%, w/v) was incubated at 37°C for 30 min and residual protease activity was estimated [23]. Further, one ml each of different concentrations (0.33, 0.66, 1.0, 2.0, 3.0 mg ml^-1^) of most effective plant seed extract fraction was incubated with 1 ml of trypsin (100 μg ml^-1^, 455 caseinolytic units), fungal protease (245 caseinolytic units) and bacterial protease (98 caseinolytic units) at 37 ± 1°C for 1 h. Following incubation, the residual protease activity of trypsin, bacterial and fungal protease was estimated as per the method described earlier [23]. The inhibition was calculated from the difference between untreated (without seed extract) and treated (with seed extract) samples divided by untreated sample reading, multiplied by 100 [27]. Protease activities of individual trypsin, fungal and bacterial proteases were taken as control. The experiment was carried out in triplicates.

#### (C) Electrophoretic analysis

The dialyzed 30-60% ammonium sulphate fraction and active elutes' fraction(s) obtained from gel filtration were subjected to 15% SDS-PAGE [28]. In another set of experiment, an activity gel having gelatin (0.1% w/v) co-polymerized with SDS-PAGE was used to analyze the PI activity [29]. The samples were not boiled prior to loading. In activity gel, after appropriate run, SDS was removed by washing the gel with triton X-100 (50 ml, 2.5% v/v) for 45 min. The gel was washed twice with distilled water and then incubated with 100 ml of trypsin (250 μg ml^-1^, 1137.5 U ml^-1^, in 100 mM Tris-HCl buffer, pH 8.0) at 37°C for 1 h. The gel was again washed with distilled water and stained using Coomassie brilliant blue R-250 stain. The active fraction was observed as distinct blue band after proper destaining.

### Antifungal activity assay

The dialyzed extract (30-60%) was used to study its effect on conidial germination of *Aspergillus flavus*. The seeds' extract (1.8 mg ml ^-1^) was filtered using nitrocellulose membrane (pore size 0.22 μm), and its 50 μl fraction was mixed with 100 μl of potato dextrose broth (PDB) containing conidia (7 × 10^-4^), poured in depression slide and incubated at 37°C for 36 h. Heat inactivated (100°C for 60 min) seed extract was used as PI control. The inhibition pattern was also confirmed using pooled active fraction obtained after SEC.

### Statistical analysis

The spot test experiments were carried out thrice each in duplicate. In the caseinolytic assay analysis, standard deviation (SD) was calculated using Microsoft Excel. Further, analysis of variance (ANOVA) was performed using GraphPad InStat version 3.10.

## Results and Discussion

### Spot-test analysis

**Figure 1:**
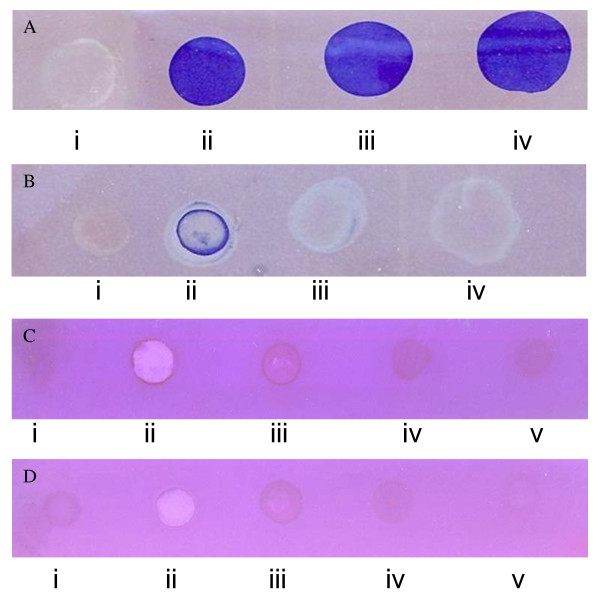
**Detection of protease inhibitory activity using X-ray film**. Trypsin (5, 10 & 15%, w/v in Tris-HCl buffer) cleared the gelatin coat on film (Figure 1A-ii, iii & iv). Buffer alone served as control (Fig. 1 A-i). Trypsin (10%), preincubated with heat-treated crude seed extract (2 μg ml^-1^) for 60 min., was moderately inhibited and partial gelatin clearance was observed (Figure 1 B-ii). At higher concentrations (5, 12.5 μg ml^-1^), trypsin was completely inhibited and no gelatin digestion was seen (Figure 1 B-iii, iv). The heat-treated plant material (no endoproteolytic activity) served as control (Fig1 B-i). The inhibitory effect against *Aspegillus flavus *and *Bacillus *sp. protease is shown in Figure 1 C & D respectively. *Aspegillus flavus *and *Bacillus *sp. protease caused gelatin digestion (Figure 1 C-ii & D-ii) which upon incubation with seed extract was not seen (Figure 1 C-iii, iv & D-iii, iv). Buffer alone (Figure 1C -i), heat treated (Figure 1D -i) and inactivated seed extract (Figure 1C -v & D -v) served as control.

Trypsin produced a distinct zone of gelatin clearance (Figure 1A) on x-ray film. After incubation with preheated plant seed extract for 1 h, trypsin exhibited variable strength of clearance with varied seed extract dilutions. At lowest extract concentration, the inhibition was partial as evident from incomplete gelatin digestion (Figure 1B-ii). However, at increasing seed extract concentrations (5 & 12.5 μg ml^-1^), such clearance was not evident (Figure 1B-iii &1iv), which is indicative of complete trypsin inhibition. Similar to trypsin, the culture supernatant of *Aspergillus flavus *(caseinolytic activity 245 U ml^-1^) and of *Bacillus *sp. (caseinolytic activity 98 U ml^-1^) also cleared the gelatin coated on x-ray film (Figure 1C-ii, 1D-ii). However, upon incubation with seed extract, both the supernatants were unable to clear the gelatin, thereby indicating their effective inhibition (Figure 1C-iii, 1iv &1D-iii, iv). These results reflect presence of strong protease inhibitor(s) in *Cassia tora *seed extract. The seed extracts used as control did not cause gelatin digestion (Figure 1C-i, 1v &1D-i, v). The spot test method is a rapid and sensitive test for visual detection of protease inhibitors even in lower quantities. Cheung *et al*. studied the effect of soybean trypsin inhibitor mixed with trypsin on X-ray film and reported that 2 μg ml^-1 ^inhibitor was effective in trypsin inhibition [30]. The x-ray film consists of acetate cellulose coated on both sides with silver halide and gelatin. Gelatin is a substrate for proteolytic enzymes, and minimum effective inhibitory concentrations reflect the sensitivity of this method.

The heat treatment of plant extract is critical for denaturation of endogenous thermolabile proteases as they may interfere with results. Gallagher *et al*. [31] have also described that endogenous protease activity of the plants can lead to artifacts in screening of inhibitors. Further, it is also established, albeit indirectly, that inhibitor fraction in *Cassia tora *seed extract is thermostable. Proteins involved in defense mechanism are known to be heat stable, and thermostability of seed extract for protease inhibition is apparently in consonance with report of *Azarkan et al*. [32]. Lopes *et al*. have reported a heat stable trypsin inhibitor from *Acacia plumose *having antifungal properties against *Aspergillus niger *and *Fusarium moniliforme *[33].

### Casein digestion (Caseinolytic) method

**Figure 2:**
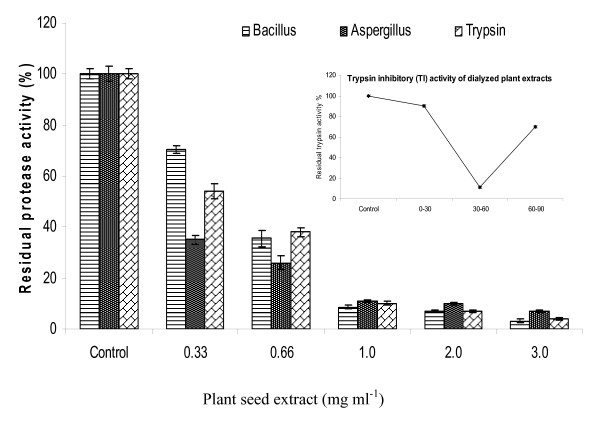
**Residual activities of trypsin, bacterial and fungal proteases in the presence of different concentrations of dialyzed seed extract (30-60%)**. One ml plant extract (0.33, 0.66, 1.0, 2.0 and 3.0 mg ml^-1^) preincubated with 1 ml each of trypsin (455 U caseinolytic activity), *Bacillus *sp. (98 U caseinolytic activity) and *Aspergillus flavus *protease (214 U caseinolytic activity). Trypsin inhibitory activity pattern of different ammonium sulphate fraction is shown in Figure 2 (inset).

Among all, the dialyzed 30-60% ammonium sulphate fraction exhibited maximum antitryptic activity, and was used for further studies (Figure 2 inset). In this fraction, the trypsin activity was progressively reduced upon incubation with increased plant seed protein extract (Figure 2). At lower concentration of protein in plant extract (0.33 and 0.66 mg ml^-1^), the residual activity was 56 and 38%, respectively, which further reduced to 10% (1 mg ml^-1 ^seed extract). The overall inhibition of trypsin increased up to 96% at higher concentration (3 mg ml^-1^). The ANOVA revealed significant P value (< 0.001), which is indicative of strong inhibitory effect of plant extract even at low concentrations. However, Tukey-Kramer Multiple comparisons test showed less significant P value (> 0.05) between 1 and 2 mg ml^-1 ^seed extract and between 2 and 3 mg ml^-1 ^extract. The pattern of protease inhibition in culture supernatant of *Aspergillus flavus *and *Bacillus *sp. was similar to trypsin. The caseinolytic activities of *Aspergillus flavus *proteases remained 35 and 26% at lower concentrations (0.33 and 0.66 mg ml^-1^). It was further reduced to 11% only at 1 mg ml^-1 ^plant protein. The *Bacillus *sp. protease activity was inhibited up to 65% and 91% at 0.66 and 1.0 mg ml^-1 ^seed extract, respectively (Figure 2). The P value (< 0.001) is significant for inhibitory effect of seed extract against fungal and bacterial proteases at all low concentrations. However, Tukey-Kramer Multiple comparisons test showed less significant P value (> 0.05) between higher concentrations of 1, 2 and 3 mg ml^-1 ^extract. In non parametric analysis of variance (Kruskal-Wallis test), the inhibitory effect of plant extract against all proteases was significant (P value 0.0162). The pattern of inhibition of proteolytic activity of trypsin corresponds with the findings of Pando *et al*. [34] and Bhattacharyya *et al*. [35]. Baker *et al*. reported an inhibitor, having limited activity against *Aspergillus flavus*, from maize displaying 84% trypsin inhibition comparable to that exhibited by soybean trypsin inhibitor [36]. Earlier, a maize trypsin inhibitor was reported as the basis of resistance against *Aspergillus flavus *infection [37].

The prevalence of *Aspergillus flavus *in plants, farm animals and above all in humans transforms it to a potent threat to agriculture and human health. Since strains of *Aspergillus flavus *lack host specialization [13], and produces virulent proteases, it leads to allergenicity (due to serine proteases), invasive aspergillosis and cutaneous infections [12]. Therefore, inhibition of their proteolytic activity is important for generalized defense response in diverse organisms.

Different species of *Bacillus *remain associated with various gastrointestinal and non-gastrointestinal infections in normal and immunocompromised individuals. The bacterium may feed on host tissues by producing toxins, phospholipases and proteases. Proteins, peptides and amino acids have been suggested as the preferred nutrient sources for *Bacillus cereus *[17], possibly linked to the growth of bacterium as a human and animal pathogen. Thus, proteases help in defense and survival of the organism in host cell environment. The attenuation of bacterial protease virulence employing biomolecules like protease inhibitor can be an effective strategy to combat such infections. This presumably provides competitive advantage to the host immune system, as ability of bacterial adaptation to the mammalian environment may be stressed or abolished. It is also expected that protease inhibitors unlike conventional antibiotics would not exert a selective pressure leading to development of resistance in pathogenic microorganisms [38].

### Electrophoretic analysis

**Figure 3:**
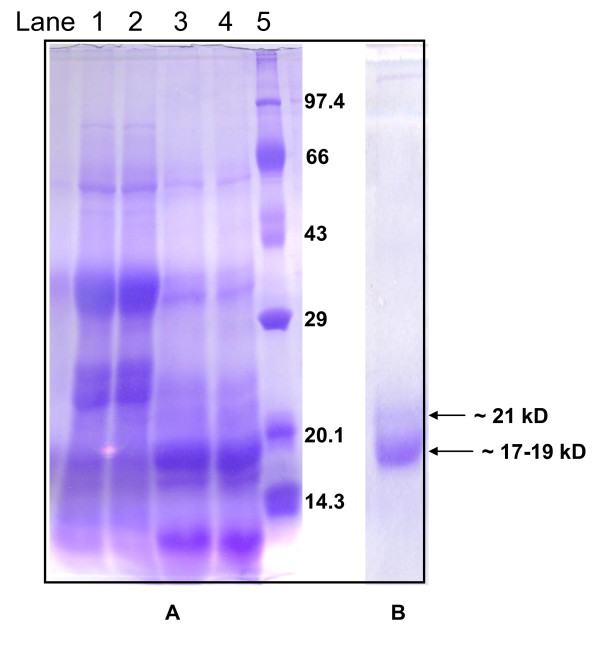
**SDS-PAGE and Activity gel for detection of trypsin inhibitory activity of plant extract**. Denaturing gel electrophoresis of 30-60% dialyzed seed extract contain many proteins of different sizes (lanes 1& 2); active fraction pooled after size exclusion chromatography (SEC) (lanes 3 & 4); lane 5, molecular weight marker (Fig. 3A). In activity gel, a major band (~17-19 kD) representing active trypsin inhibitor(s) is evident (Fig. 3B).

In the present study, the denaturing and gelatin SDS-PAGE analysis revealed activity in seed extract against trypsin. Previous studies indicated the presence of most active inhibitor in 30-60% ammonium sulphate fraction. Though, 30-60% fraction contained many other proteins (lane 1&2, Figure 3A), they were digested upon incubation with trypsin, and a major band (~17-19 kD) of protein(s) indicating resistance to tryptic digestion was left (Figure 3B). However, probably on account of imperfect denaturation (sample not boiled) an additional band of ~21 kD is also visible which is not present in the corresponding SDS-PAGE (Figure 3B). The dark blue band (formed due to complex of nonhydrolyzed gelatin and plant protein) presumably represents major proteinaceous protease inhibitor(s) of *Cassia tora *seed. The fraction obtained from gel filtration also displayed the same active protein(s) in activity gel (not shown). The above active protein band(s) coincide with (lane 5, Figure 3A) the range of molecular weight (~20 kD) characteristic of Kunitz type protease inhibitors [39]. Felicioli *et al*. stressed the potential of gelatin containing polyacrylamide gel electrophoresis in visualization of protein inhibitors [40].

### Antifungal activity assay

**Figure 4:**
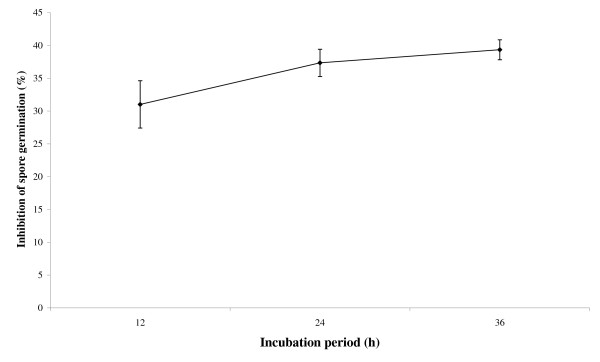
**Inhibition of *Aspergillus flavus *conidia germination**. Ammonium sulphate fraction (50 μl, 30-60%) mixed with 100 μl of potato dextrose broth (containing 7 × 10^-4 ^fungal conidia) exhibited moderate inhibition of spore germination on 36 h incubation at 37°C.

The assessment of antifungal activity by inhibition of fungal conidia germination in potato dextrose broth (PDB) at 24^th ^h revealed that spore germination was moderately reduced. The 50 μl of plant extract in conidial suspension resulted in inhibition of about 30% conidia in 12 h. There was no further significant increase in inhibition of total conidial germination after 24 h onwards (Figure 4). Chen *et al*. reported rupture of 45% *Aspergillus flavus *conidia upon incubation with 14 kD maize trypsin inhibitor [41]. The cellular factors including proteinases produced by *Aspergillus flavus *may aid in germination of conidia and pathogenesis in lung alveoli cells [12]. Therefore, the proteinase inhibitors may offer relief on this account. The purified maize trypsin inhibitor simultaneously affected the growth of *Aspergillus flavus *and *Fusarium moniliforme *coexisting on maize plant as pathogens. Peng and Black concluded in their studies that proteinase inhibitory activity in resistant tomato plant was raised in response to infection by *Phytopthora infestans *[42].

## Conclusions

The results revealed an unreported protease inhibitory activity in *Cassia tora *seeds. Traditionally, the plant is recommended in skin and gastrointestinal disorders. Inhibition of trypsin, bacterial and fungal proteases is indicative of its possible mechanism for varied therapeutic applications. The denaturing and activity gel electrophoresis revealed common protein(s) present in seed responsible for anti-tryptic activity. *Aspergillus flavus *with potential to affect crop yield and human health by aflatoxins can be attenuated by employing virulence attenuators like protease inhibitors. The *Cassia tora *seed extract moderately inhibited the spore germination of *Aspergillus flavus*. This can also be effectively used to bolster the plant defense response by employing biotechnological techniques in crop protection.

## Competing interests

The authors declare that they have no competing interests.

## Authors' contributions

VRT carried out the isolation, spot test, caseinolytic and electrophoretic studies. SK designed and participated in antifungal activity assay. VRT and SK drafted the manuscript. SKG coordinated the design of work, revised the manuscript critically. All authors read and approved the final manuscript.

## Pre-publication history

The pre-publication history for this paper can be accessed here:

http://www.biomedcentral.com/1472-6882/11/56/prepub
